# *Pien-Tze-Huang* attenuates neuroinflammation in cerebral ischaemia-reperfusion injury in rats through the TLR4/NF-κB/MAPK pathway

**DOI:** 10.1080/13880209.2021.1942926

**Published:** 2021-07-01

**Authors:** Xiaoqin Zhang, Qing Zhang, Lili Huang, Mingzhen Liu, Zaixing Cheng, Yanfang Zheng, Wen Xu, Jinjian Lu, Jian Liu, Mingqing Huang

**Affiliations:** a College of Pharmacy, Fujian Key laboratory of Chinese Materia Medica, Fujian University of Traditional Chinese Medicine, Fuzhou, China; b State Key Laboratory of Quality Research in Chinese Medicine, Institute of Chinese Medical Sciences, University of Macau, Macao, China

**Keywords:** Ischaemic stroke, neuroprotection, signalling pathways

## Abstract

**Context:**

*Pien-Tze-Huang* (PTH) is traditionally applied to treat various inflammation-related diseases including stroke. However, literature regarding the anti-inflammatory effects and possible mechanisms of PTH in ischaemic stroke is unavailable.

**Objective:**

This study investigates the anti-inflammatory effects and its underlying mechanism of PTH on ischaemic stroke.

**Materials and methods:**

Cerebral ischaemia-reperfusion injury was induced through 2 h middle cerebral artery occlusion (MCAO) followed by 24 h reperfusion in male Sprague-Dawley (SD) rats receiving oral pre-treatment with PTH (180 mg/kg) for 4 days. TLR4 antagonist TAK-242 (3 mg/kg) was injected intraperitoneally at 1.5 h after MCAO. MRI, HE staining, qRT-PCR, western blot, and immunofluorescence methods were employed.

**Results:**

PTH treatment markedly reduced cerebral infarct volume (by 51%), improved neurological function (by 33%), and ameliorated brain histopathological damage in MCAO rats. It also reduced the levels of four inflammatory mediators including IL-1β (by 70%), IL-6 (by 78%), TNF-α (by 60%) and MCP-1 (by 58%); inhibited microglia and astrocyte activation; and decreased protein expression of iNOS and COX-2 in injured brains. Moreover, PTH down-regulated the protein expressions of TLR4, MyD88, and TRAF6; reduced the expression and nuclear translocation of NF-κB; and lowered the protein expressions of p-ERK1/2, p-JNK, and p-p38. Similar effects were observed in MCAO rats with TAK-242 treatment. However, combined administration of PTH and TAK-242 did not significantly reinforce the anti-inflammatory effects of PTH.

**Discussion and conclusion:**

PTH improved cerebral ischaemia-reperfusion injury by inhibiting neuroinflammation partly via the TLR4/NF-κB/MAPK signalling pathway, which will help guide its clinical application.

## Introduction

Ischaemic stroke, a common and terrible disease worldwide, often results in high rates of death and disability (Benjamin et al. 2018). It occurs when the cerebral artery is suddenly blocked followed by a series of pathological events, such as inflammatory response, calcium influx, excitatory toxicity, oxidative stress, and cell apoptosis (Ostrowski et al. 2016; Gonzalo-Gobernado et al. 2019). Currently, available therapeutic agents for this disease are limited and exhibit poor clinical outcomes. Among these limited agents, tissue plasminogen activator is the only one approved by the FDA; however, its clinical application is severely restricted due to its narrow therapeutic window and high risk of intracerebral haemorrhage (Berekashvili et al. 2018; Bai et al. 2019). Therefore, therapeutic agents with increased effectiveness and safety are urgently needed for preventing or treating ischaemic stroke.

Accumulating evidence shows that inflammation plays a crucial role in different periods of ischaemic stroke, which not only results in brain injury but is also closely associated with functional recovery (Tuttolomondo et al. 2009; Tobin et al. 2014). Toll-like receptors (TLRs), especially Toll-like receptor 4 (TLR4), play an integral part in the inflammatory cascade reaction after cerebral ischaemia-reperfusion injury (Beutler 2009; Lavelle et al. 2010). TLR4 is swiftly stimulated by various endogenous ligands (Parada et al. 2019) and subsequently activates protein myeloid differentiation primary response gene 88 (MyD88) after cerebral ischaemia (Kumar 2019). As a result, two important signalling pathways, namely nuclear factor-κB (NF-κB) and mitogen-activated protein kinases (MAPKs), are activated to produce proinflammatory cytokines and further aggravate tissue damage (Tse et al. 2014). In addition, the inhibition or knockout of TLR4 can attenuate inflammatory responses and protect against ischaemic brain injury in rodent (Shichita et al. 2012; Denlinger 2016). Thus, targeting the TLR4/NF-κB/MAPK signal pathway and its mediated inflammation is a potential therapeutic direction against ischaemic stroke.

Traditional Chinese medicine (TCM) has attracted extensive attention for preventing and treating cerebrovascular diseases based on its unique theoretical system and comprehensive therapeutic effects. As one of the most famous TCM preparations in China, *Pien-Tze-Huang* (PTH) is composed of Radix et *Rhizoma Notoginseng*, Moschus, Calculus Bovis, and Snake Gall, which was first prescribed in 1555 AD. It is commonly applied to treat various inflammation-related diseases including stroke attributed to its traditional clearing fever and detoxifying, namely anti-inflammatory effect (Huang et al. 2019). There are several pharmacological studies investigated the protective and therapeutic effects of PTH against ischaemic stroke, which confirmed its traditional application in stroke therapy (Zhang et al. 2010, 2011). Our recent study demonstrated that pre-treatment with 180 mg/kg PTH can decrease infarct volume and alleviate neurological deficits in MCAO rats by inhibiting the mitochondria-mediated apoptotic pathway and modulating the protein kinase B/glycogen synthase kinase-3β pathway (Zhang et al. 2018). Importantly, the up-regulated inflammatory cytokines including tumour necrosis factor-α (TNF-α), interleukin (IL)-6 and IL-1β in injured brain were also remarkably suppressed by PTH, indicating that the anti-inflammatory effect may play part in the anti-stroke potency of PTH (Liu et al. 2014; Zhang et al. 2018). Moreover, recent studies have revealed the anti-inflammatory effects of PTH in hepatitis through regulating NF-κB pathway (Zheng et al. 2019; Deng et al. 2020). However, the detailed relationship between the anti-inflammatory and anti-stroke effects of PTH remains unclear.

Collectively, we hypothesised that the effect of PTH on cerebral ischaemia-reperfusion injury is ascribed to suppression of neuroinflammation. In the present study, MCAO rats were applied to explore whether PTH attenuate neuroinflammation in cerebral ischaemia-reperfusion, and to further illustrate the possible molecular mechanisms involved in the TLR4/NF-κB/MAPK signalling pathway.

## Materials and methods

### Reagents

TAK-242 (HY-11109) was obtained from MedChemExpress (Monmouth Junction, NJ, USA). TLR4 (sc-293072), MyD88 (sc-74532), inhibitor of NF-κB α (IκBα) (sc-1643), and NF-κB p65 (sc-8008) primary antibodies were provided by Santa Cruz Biotechnology (Dallas, TX, USA). cyclooxygenase 2 (COX-2) (12282), glial fibrillary acidic protein (GFAP) (3670), p38 (8690), c-Jun N-terminal kinase (JNK) (9252), extracellular signal-regulated kinase 1/2 (ERK1/2) (9102), p-ERK1/2 (Thr202/Tyr204) (9101), and glyceraldehyde-3-phosphate dehydrogenase (GAPDH) (2118) primary antibodies were provided by Cell Signalling Technology (Danvers, MA, USA). inducible nitric oxide synthase (iNOS) (ab3523) and TNF receptor-associated factor 6 (TRAF6) (ab33915) primary antibodies were provided by ABCAM (Cambridge, MA, USA). Each secondary antibody was provided by Thermo Fisher Scientific (Rockland, IL, USA).

PTH capsules were purchased from Zhangzhou PTH Pharmaceutical Co., LTD. (Zhangzhou, China). In accordance with our reported UPLC-MS/MS method (Huang et al. 2016), 21 characteristic compounds (taurine, malic acid, citric acid, cholic acid, hyodeoxycholic acid, ursodeoxycholic acid, chenodeoxycholic acid, taurocholic acid, taurochenodeoxycholic acid, tauroursodeoxycholic acid, glycodeoxycholic acid, glycocholic acid, notoginsenoside R1, ginsenosides Rg1, Rb1, Re, Rf, Rd, Rg2, Rg3, Rh1, and muscone) in the PTH capsules were accurately quantified, and the total content of those compounds was 183.73 mg/g.

### Animals

Male Sprague-Dawley (SD) rats weighing 230–260 g were provided by the Guangdong Experimental Animal Centre (Guangzhou, no. SYXK-2019-0007). Animals were provided food and water *ad libitum* under SPF conditions at the temperature of 24 °C ± 1 °C and relative humidity of 60% ± 5%. All animal experiments were conducted with the approval of the Animal Care and Use Committee of Fujian University of Traditional Chinese Medicine.

### Preparation of the Middle cerebral artery occlusion model

The transient middle cerebral artery occlusion (MCAO) model was established in accordance with our previous protocol (Longa et al. 1989). Briefly, male SD rats were anaesthetized with 5% isoflurane (Shenzhen, China) and maintained with 2% isoflurane. The left common, external, and internal carotid arteries were separated. A 0.38 mm nylon suture with a L3800 silicon-coated tip (Guangzhou, China) was carefully inserted into the internal carotid artery from the external carotid artery to occlude the middle cerebral artery. After occlusion for 1.5 h, the nylon suture was withdrawn to induce reperfusion. Sham rats were subjected to the same procedure without inserting the nylon suture into the MCAO.

### Drug administration

Experiment 1: After adaption for 7 days, the rats were randomly allocated to three groups (*n* = 12): (1) Sham group: sham rats intragastrical (i.g.) administered 0.5% CMC-Na (10 mL/kg); (2) MCAO group, MCAO rats i.g. administered 0.5% CMC-Na (10 mL/kg); (3) MCAO + PTH group, MCAO rats i.g. administered PTH (10 mL/kg). PTH was dissolved in 0.5% CMC-Na (18 mg/mL) and administered at the dose of 180 mg/kg once a day for 4 days before MCAO as previously described (Zhang et al. 2018).

Experiment 2: After adaption for 7 days, animals were randomly divided into five groups (*n* = 6): (1) Sham group: sham rats i.g. administered 0.5% CMC-Na; (2) MCAO group, MCAO rats i.g. administered 0.5% CMC-Na; (3) MCAO + PTH group, MCAO rats i.g. administered PTH (180 mg/kg); (4) MCAO + TAK-242 group, MCAO rats intraperitoneal (i.p.) administered TAK-242 (3 mg/kg); (5) MCAO + PTH + TAK-242 group, MCAO rats i.g. administered PTH (180 mg/kg) and i.p. administered TAK-242 (3 mg/kg). TAK-242, an antagonist of TLR4, was dissolved in 10% DMSO and administered at the dose of 3 mg/kg at 1.5 h after MCAO (Hua et al. 2015). The other experimental conditions were the same as those described above.

### Neurological deficit evaluation

In accordance with the reported procedures and criteria (Zhang et al. 2018), the neurological deficit condition of each rat was evaluated blindly at 24 h after reperfusion.

### Infarct volume measurement

Magnetic resonance imaging (MRI) test was used to determine the infarct volume as follows: A rat was anaesthetised with 5% isoflurane and fixed in the animal cradle with its head inside the horizontal magnet bore of a BioSpec 70/20 USR 7.0 T MRI scanner (Bruker BioSpin, Ettlingen, Germany). Then, the coronal plane image was prescribed beginning at 3 mm behind the olfactory bulb. T2 weighted imaging scans were performed with a turbo-rapid acquisition relaxation enhancement sequence. All images were obtained with the repetition time of 4200 ms, echo time of 55 ms, field of view of 32 × 32, matrix size of 256 × 256, slice thickness of 1 mm, and number of slices of 21. Image J software was used to calculate the infarct volume.

### Haematoxylin–eosin (HE) staining

Each rat was anaesthetised with 5% isoflurane, and its heart was perfused successively with saline and 4% paraformaldehyde. Then, its brain was removed, fixed, dehydrated, and embedded. The brain was cut into 5 µm thick sections and stained with haematoxylin and eosin (Beyotime, Shanghai, China). Finally, the sections were photographed under a DMi8 light microscope (Leica, Wetzlar, Germany).

### Quantitative real-time polymerase chain reaction (qRT-PCR) analysis

Ischaemic cerebral hemisphere was removed from deeply anaesthetised rats at 24 h after reperfusion. Total RNA was extracted by using Trizol (Invirtogen) and reverse transcribed into cDNA with a Superscript First-Strand Synthesis System (Life Technologies, Grand Island, NY). Quantitative analysis was performed by using SYBR Green real-time PCR master mix (Life Technologies). GAPDH was used as the internal control in this study. The PCR primer sequences are listed in Table 1.

**Table 1.** Primer sequences of IL-1β, IL-6, TNF-α, MCP-1 and GAPDH.

Gene	Sense (5’-3’)	Anti-sense (5’-3’)	Length (bp)
IL-6	GACTTCCAGCCAGTTGCCTT	CTGGTCTGTTGTGGGTGGTAT	112
IL-1β	CTGTCTGACCCATGTGAGCTG	TTTGTCGTTGCTTGTCTCTCCTT	108
TNF-α	ATGGGCTCCCTCTCATCAGT	GCTTGGTGGTTTGCTACGAC	106
MCP-1	CAGGTCTCTGTCACGCTTCT	GTAGTTCTCCAGCCGACTCA	150
GAPDH	CAACGGGAAACCCATCACCA	ACGCCAGTAGACTCCACGACAT	96

### Immunofluorescent staining

Each brain was removed and embedded in paraffin as described above. After dewaxing and rehydration, each brain section (5 µm) was subjected to antigen retrieval solution for 15 min in a microwave oven and then blocked with 5% BSA containing 10% goat serum for 2 h. It was incubated overnight at 4 °C with mouse monoclonal anti-NF-κB p65 antibody (1:100) or anti-Iba-1 antibody (1:300) diluted in the blocking solution. Subsequently, it was washed with PBST and incubated with FITC-conjugated goat anti-mouse IgG (1:200) for 1 h at 25 °C. The nuclei were counterstained with DAPI (Beyotime) and cover-slipped. Finally, the immunofluorescent images were captured under a DMi8 microscope (Leica) and magnification of 200×. The number of NF-κB p65 or Iba-1-positive cells in five similar fields of the ipsilateral cortex from three sections per rat was counted by an investigator who was blinded to the treatment.

### Western blot analysis

Proteins were extracted from ischaemic cerebral hemispheres by using RIPA lysis buffer. BCA Protein Assay kit (Beyotime, Shanghai, China) was used to measure the protein concentration of each sample. Western blot analysis was conducted in accordance with our previous study (Zhang et al. 2018). In brief, each protein sample (30 µg) was separated through SDS-PAGE then transferred and blocked. Primary antibodies against TLR4 (1:500), MyD88 (1:500), TRAF6 (1:5000), IκBα (1:500), NF-κB p65 (1:500), p38 (1:1000), p-p38 (1:1000), JNK (1:1000), p-JNK (1:1000), ERK1/2 (1:1000), p-ERK1/2 (1:1000), COX-2 (1:1000), iNOS (1:500), GFAP (1:1000), and GAPDH (1:1000) were used. GAPDH was used as a control. Images were visualised by using ChemiDoc XRS + imaging system (Bio-Rad, Hercules, CA), and the target bands were scanned and analysed quantitatively by using Image J software.

### Statistical analysis

Data are expressed as means ± SEM and analysed with SPSS software (version 20.0). One-way AVONA was used when the data conformed to normal distribution, whereas Kruskal-Wallis test or Mann-Whitney test was used when the data conformed to non-normal distribution. *p* < 0.05 was defined as statistically significant.

## Results

### PTH decreased cerebral infarct volume and improved neurological deficits in MCAO rats

**Figure 1.**
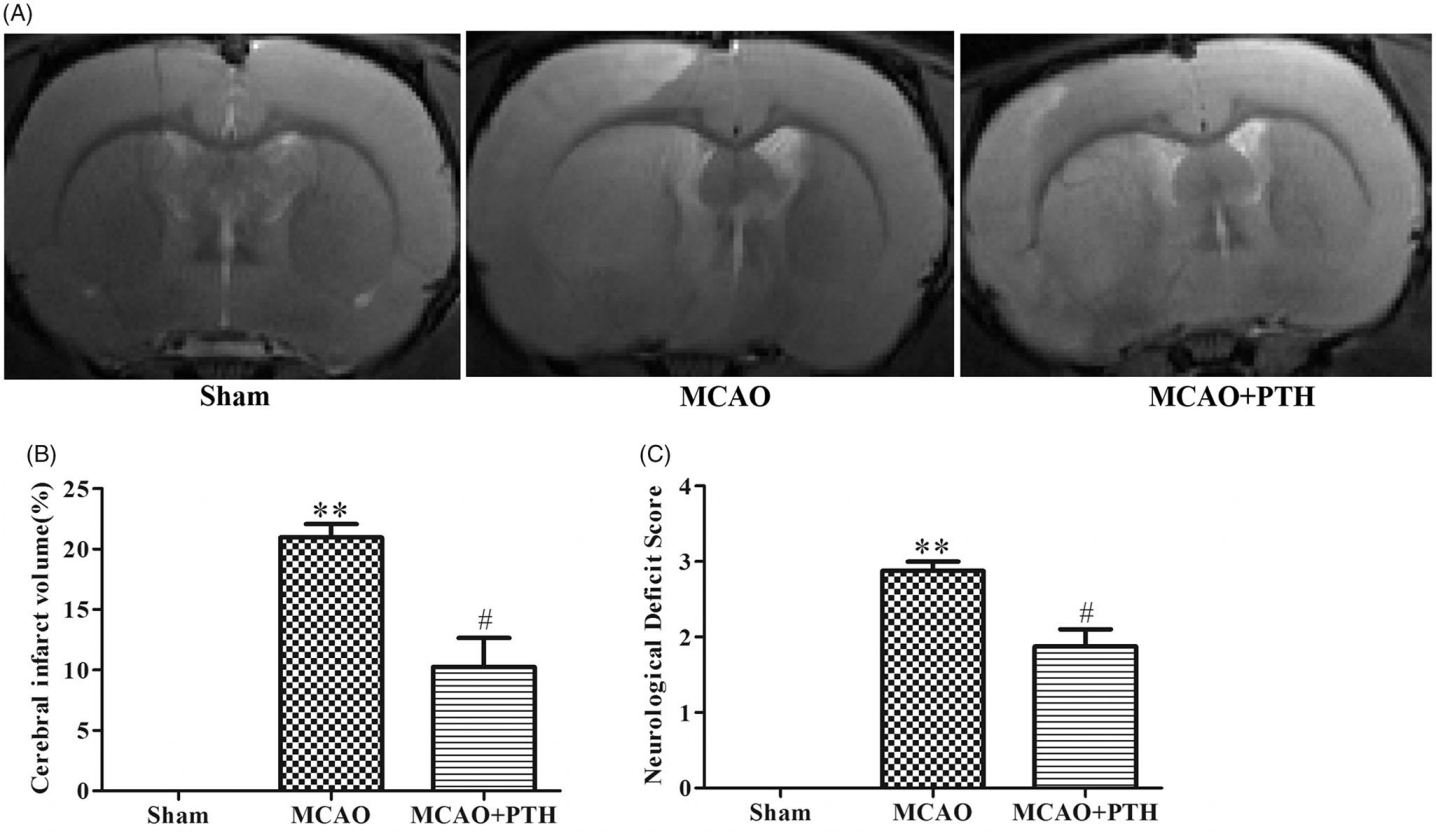
Effects of PTH on infarct volume and neurological deficits in MCAO rats. (A) Representative T2-weighted images of brain sections in different groups (*n* = 6). (B) Quantitative analysis of infarct volume in different groups (*n* = 6). (C) Quantitative analysis of neurological deficits in different groups (*n* = 6). Data are presented as mean ± SEM. **p < 0.01 *vs.* Sham; ^#^*p* < 0.05 *vs.* MCAO.

MRI results (Figure 1(A,B)) showed that relative to the Sham treatment, MCAO modelling induced remarkable increases in the cerebral infarct volume (20.97%, *p* < 0.01), whereas PTH treatment significantly reduced cerebral infarct volume (10.27%, *p* < 0.05). Similarly, as shown in Figure 1(C), MCAO modelling also resulted in worse neurological deficit compared with the Sham treatment (2.79, *p* < 0.01), and this deficit was markedly improved after PTH administration (1.86, *p* < 0.05). These data indicated that PTH could alleviate ischaemia-induced brain injury in MCAO rats.

### PTH ameliorated brain histopathological damage in MCAO rats

**Figure 2.**
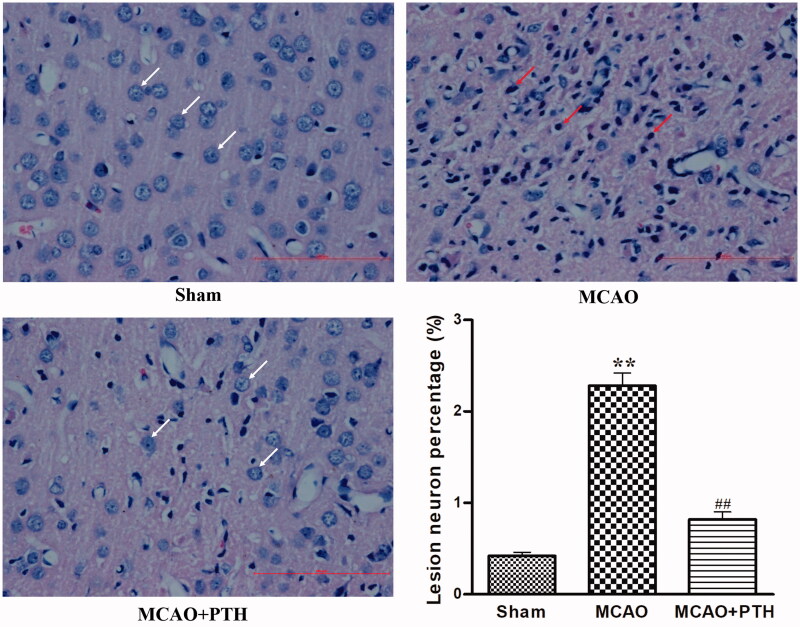
Effects of PTH on the cerebral histopathological changes in MCAO rats (*n* = 6). Haematoxylin-eosin-stained slides of the brain sections in different groups were examined under a light microscope. Normal neurons were arranged in a regular manner with intact structure (white arrow). Abnormal neurons exhibited obvious disordered arrangement, nuclei shrinkage and dark staining (red arrow). Scale bar = 100 μm.

HE staining results (Figure 2) showed that the cortical neurons of the Sham group were arranged regularly and had clear, well-stained structures, indicating the absence of histopathological changes. Compared with those of the Sham group, the cortical neurons of the MCAO group exhibited obviously disordered arrangement, nuclear shrinkage, and dark staining. However, PTH administration significantly attenuated the pathological changes induced by cerebral ischaemic injury.

### PTH down-regulated inflammatory mediators in MCAO rats

**Figure 3.**
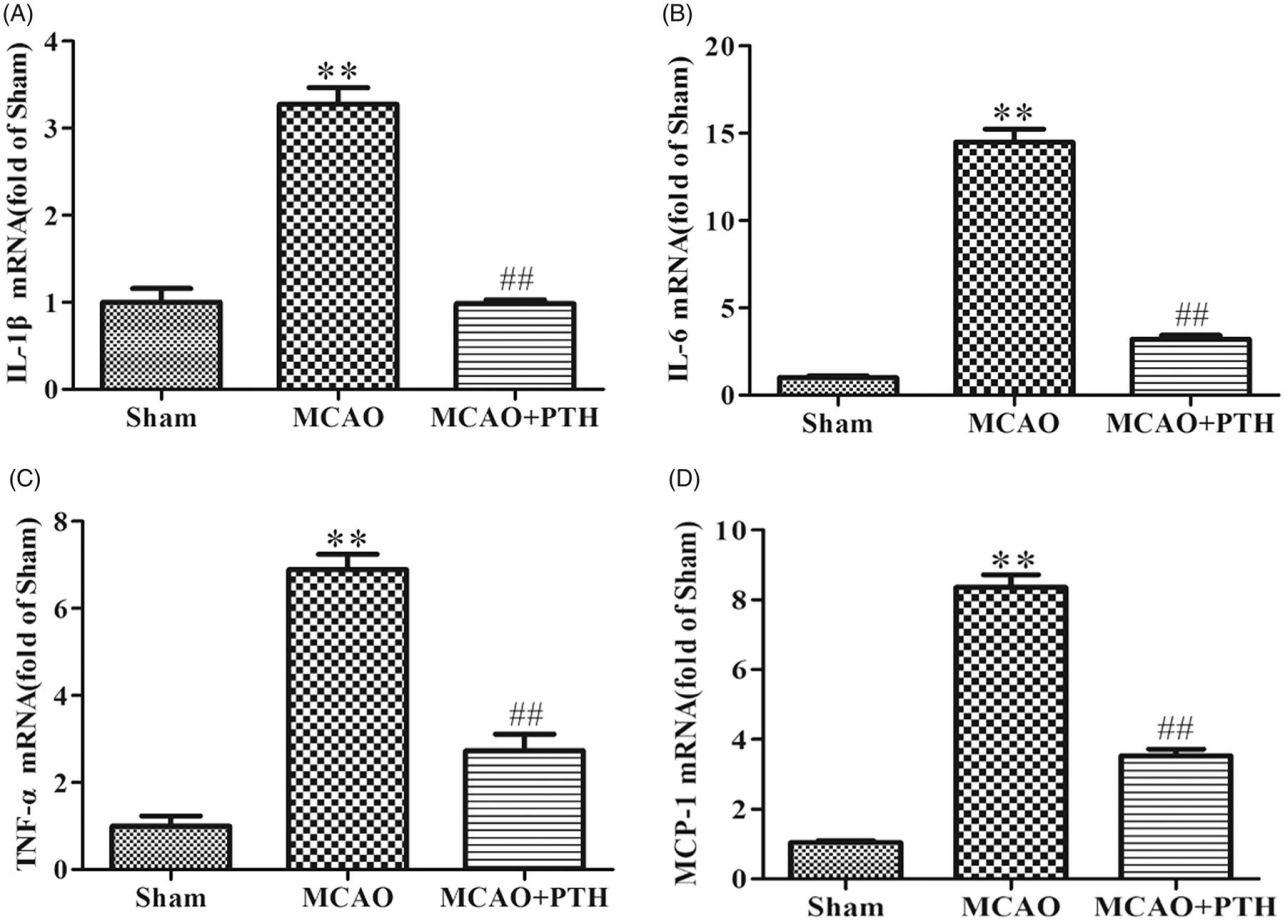
Effects of PTH on cerebral IL-1β (A), IL-6 (B), TNF-α (C), and MCP-1 (D) mRNA levels in MCAO rats (*n* = 3). Data are presented as mean ± SEM. **p < 0.01 *vs.* Sham; ^##^*p* < 0.01 *vs.* MCAO.

qRT-PCR results (Figure 3(A–D)) showed that the mRNA expression levels of three proinflammatory cytokines (IL-1β, IL-6, and TNF-α) and one chemokine (MCP-1) in the brains of the MCAO group were distinctly elevated relative to those in the brains of the sham group (*p* < 0.01). Conversely, the levels of these inflammatory mediators in the brains of the MCAO + PTH group were significantly inhibited compared with those in the brains of the MCAO group (*p* < 0.01).

### PTH inhibited microglia and astrocyte activations, and decreased iNOS and COX-2 protein levels in MCAO rats

**Figure 4.**
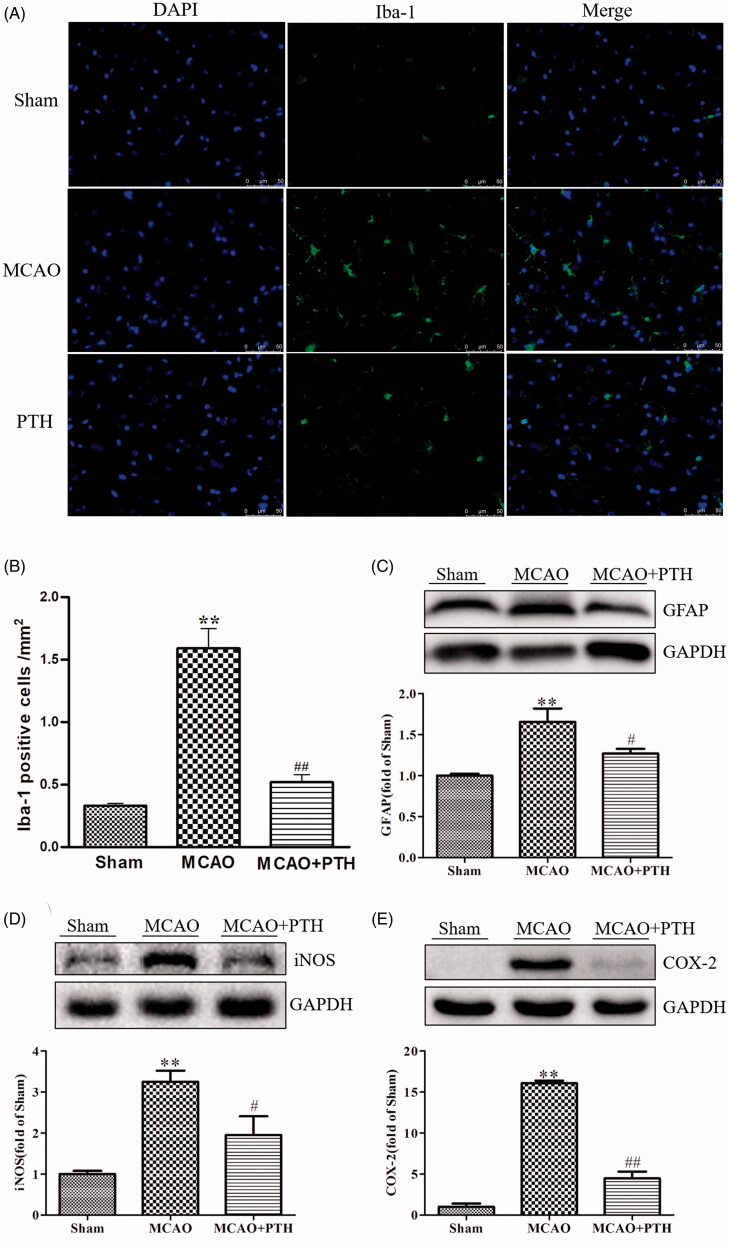
Effects of PTH on the number of Iba-1 positive cells and the protein expressions of GFAP, iNOS and COX-2 in MCAO rats (*n* = 3). (A) Representative Iba-1-staining brain sections in different groups (*n* = 3). (B) Quantitative analysis of Iba-1 positive cells in different groups (*n* = 3). (C-E) Representative western blot and quantitative analysis of GFAP (C), iNOS (D) and COX-2 (E) protein expression in different groups (*n* = 3). Data are presented as mean ± SEM. **p < 0.01 *vs.* Sham; ^#^*p* < 0.05, ^##^*p* < 0.01 *vs.* MCAO.

To further investigate the effects of PTH on neurinflammation in MCAO rats, the levels of Iba-1 and GFAP, the respective microglia- and astrocyte-specific markers, as well as two inflammatory proteins iNOS and COX-2 in the brain were measured in this study. Immunofluorescent staining results (Figure 4(A,B)) demonstrated that Iba-1 positive cells in the MCAO group were markedly increased relative to that in the Sham group (*p* < 0.01). PTH treatment significantly decreased Iba-1 positive cells (*p* < 0.01). Moreover, GFAP protein level in the MCAO group was significantly increased relative to that in the Sham group (*p* < 0.01), whereas PTH treatment significantly suppressed its expression (*p* < 0.05). These data indicated that PTH could inhibit ischaemia-induced microglia and astrocyte activations in MCAO rats.

Furthermore, as shown in Figure 4(D,E), rats in the MCAO group showed higher levels of iNOS and COX-2 in the brain tissue than rats in the Sham group (*p* < 0.01), and MCAO rats treated with PTH presented lower levels of iNOS (*p* < 0.05) and COX-2 (*p* < 0.01), further verifying the prominent anti-inflammatory effects of PTH against ischaemic stroke.

### PTH suppressed the TLR4 signal pathway

**Figure 5.**
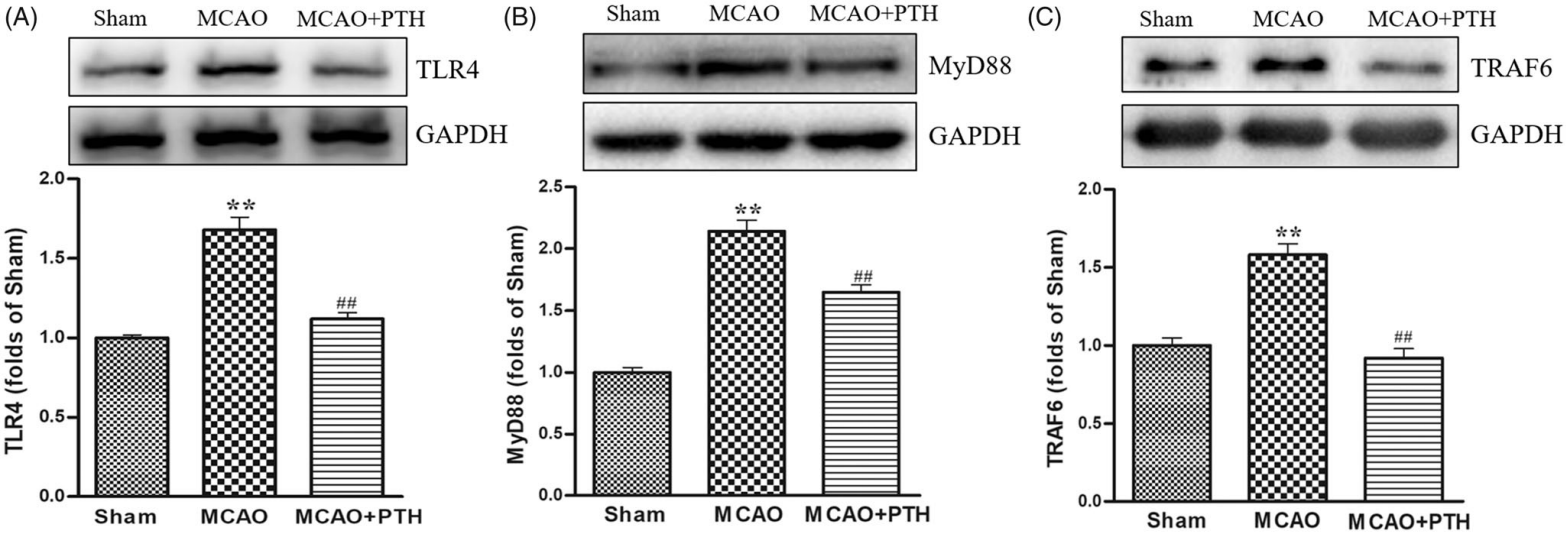
Effects of PTH on TLR4 (A), MyD88 (B), and TRAF6 (C) protein expression in MCAO rats (*n* = 3). Data are presented as mean ± SEM. *p < 0.05, **p < 0.01 *vs.* Sham; ^#^*p* < 0.05, ^##^*p* < 0.01 *vs.* MCAO.

We measured the protein levels of several key proteins in the TLR4 pathway, namely, TLR4, MyD88, and TRAF6, to investigate the possible mechanism of PTH in inflammatory responses. Western blot analysis results (Figure 5(A–C)) revealed that TLR4, MyD88, and TRAF6 protein levels were significantly elevated in the MCAO group (*p* < 0.05 or *p* < 0.01), whereas PTH treatment markedly reduced the elevated levels of these proteins (*p* < 0.05 or *p* < 0.01).

### PTH inhibited the NF-κB and MAPK signalling pathways

**Figure 6.**
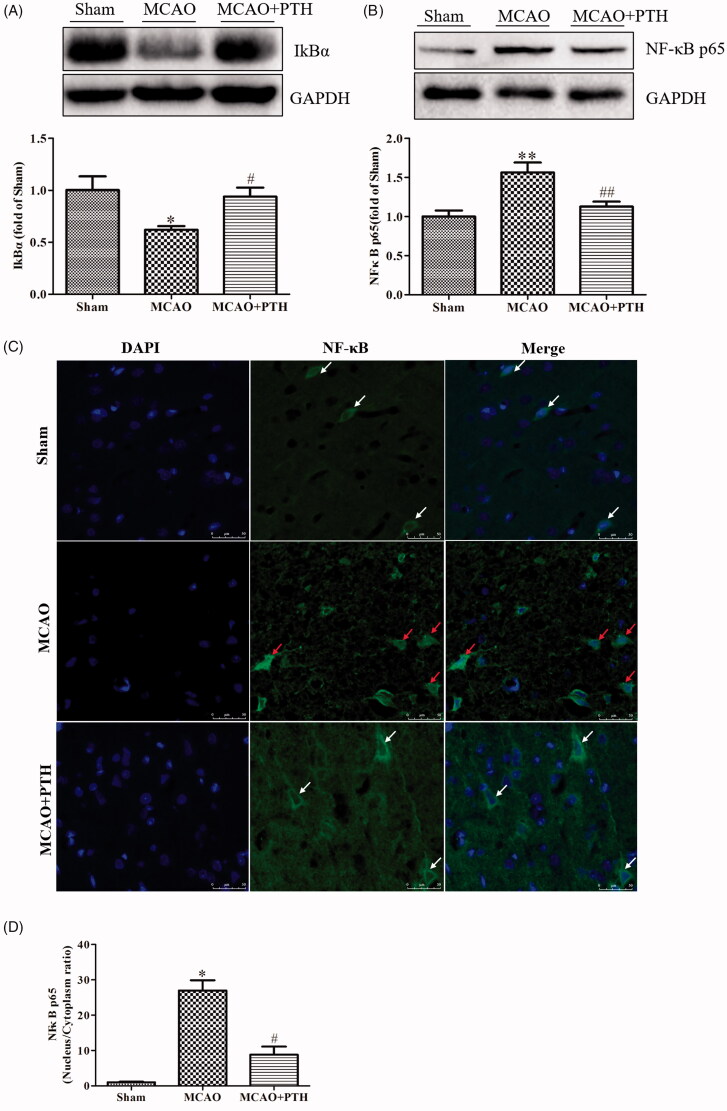
Effects of PTH on the NF-κB p65 pathway in MCAO rats (*n* = 3). (A) Representative western blot and quantitative analysis of IkBα in different groups. (B) Representative western blot and quantitative analysis of NF-κB p65 in different groups. (C) Representative immunofluorescent staining sections in different groups. (D) Quantitative analysis of the nucleus/cytoplasm ratio of NF-κB p65 in different groups. The cytosolic/nuclear translocation of NF-κB p65 was examined using immunofluorescent staining (magnification, ×200). NF-κB p65 of the normal neurons was expressed in the cytoplasm (white arrow). NF-κB p65 of the abnormal neurons was highly expressed in the nucleus (red arrow). Data are presented as mean ± SEM. *p < 0.05, **p < 0.01 *vs.* Sham; ^#^*p* < 0.05, ^##^*p* < 0.01 *vs.* MCAO. Scale bar = 50 μm.

The expression levels of several key proteins in the NF-κB and MAPK pathways were determined to further observe the effect of PTH on the downstream pathways of the TLR4 pathway. Results (Figure 6(A,B)) showed that compared with those in control rats, the NF-κB p65 level was increased and the IκBα level was decreased significantly in MCAO rats (*p* < 0.05). These changes were obviously inhibited by PTH treatment (*p* < 0.05 and *p* < 0.01). Meanwhile, we also examined the cytosolic/nuclear translocation of NF-κB p65 by using immunofluorescent staining. As illustrated in Figure 6(C,D), NF-κB p65 was highly expressed in the cytoplasm (green) in the Sham group and was significantly translocated to the nucleus in the MCAO group (*p* < 0.05). However, the nucleus/cytoplasm ratio of NF-κB p65 remarkably decreased after PTH treatment (*p* < 0.05).

**Figure 7.**
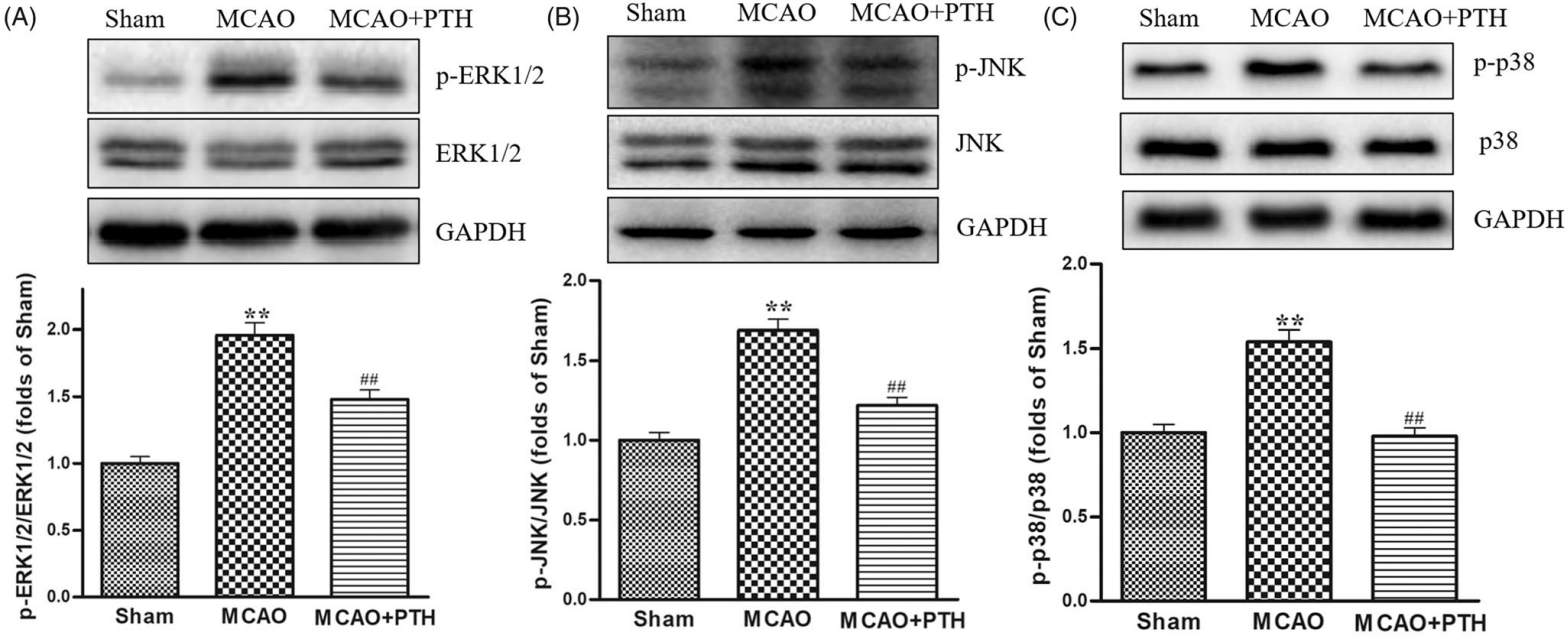
Effects of PTH on p-ERK1/2/ERK1/2 (A), p-JNK/JNK (B), and p-p38/p-38 (C) protein expression in MCAO rats (*n* = 3). Data are presented as mean ± SEM. **p < 0.01 *vs.* Sham; ^#^*p* < 0.05, ^##^*p* < 0.01 *vs.* MCAO.

Moreover, the protein levels of p-ERK1/2, p-JNK, and p-p38 in the MCAO group had significantly increased compared with those in the Sham group (Figure 7(A–C), *p* < 0.01) but were significantly down-regulated under PTH treatment (*p* < 0.05 or *p* < 0.01).

### TAK-242 did not obviously reinforce the anti-inflammatory effects of PTH on cerebral ischaemic injury

**Figure 8.**
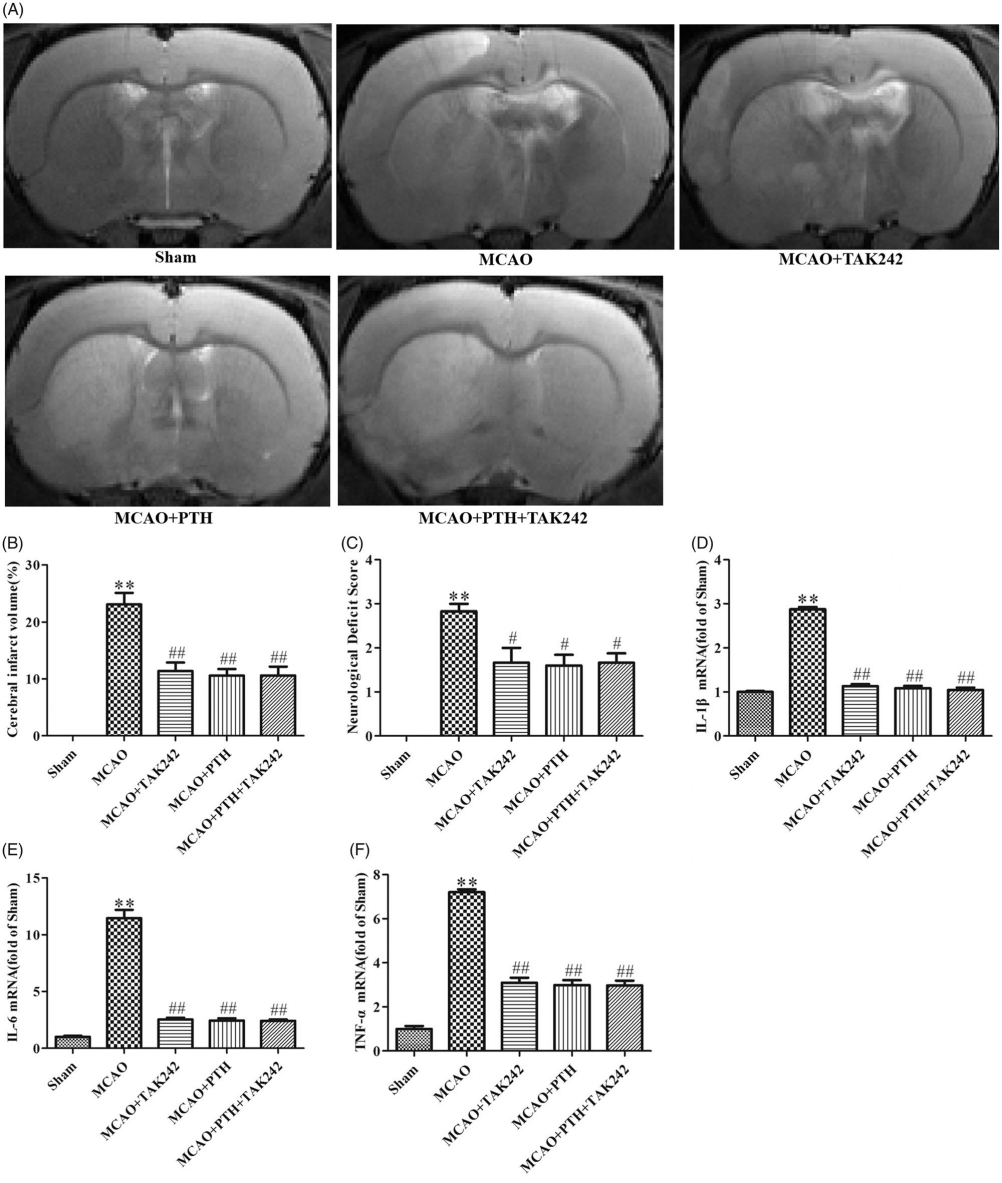
TAK-242 did not obviously enhance the anti-inflammatory effect of PTH on cerebral ischaemic injury. (A) Representative T2-weighted images of brain sections in different groups (*n* = 6). (B) Quantitative analysis of infarct volume in different groups (*n* = 6). (C) Quantitative analysis of neurological deficits in different groups (*n* = 6). (D-F) Quantitative analysis of IL-1β (D), IL-6 (E) and TNF-α (F) mRNA levels in different groups (*n* = 3). Data are presented as mean ± SEM. **p < 0.01 *vs.* Sham; ^#^*p* < 0.05, ^##^*p* < 0.01 *vs.* MCAO.

As shown in Figure 8, combined treatment with PTH and TAK-242 markedly decreased cerebral infarct volume (*p* < 0.01) and reduced neurological deficits (*p* < 0.05, Figure 8(C)). These effects were similar to the effects of PTH or TAK-242 treatment (*p* > 0.05). Moreover, the mRNA levels of IL-1β, IL-6, and TNF-α were markedly down-regulated in the groups treated with PTH, TAK-242, and PTH + TAK-242 relative to those of the MCAO group (*p* < 0.01). However, no significant difference was observed between these groups (*p* > 0.05).

**Figure 9.**
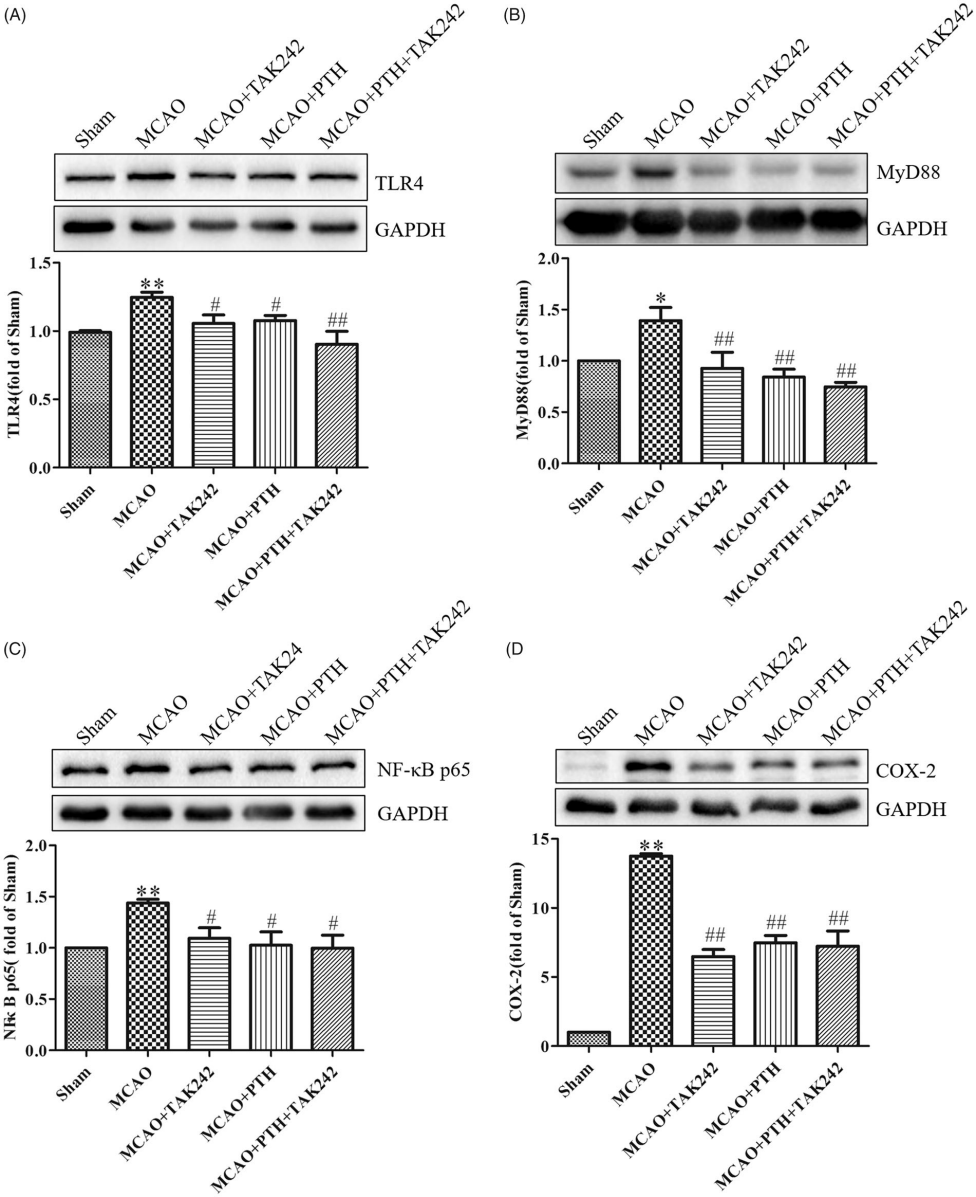
Combined administration of PTH and TAK-242 did not enhance the effects of PTH in MCAO rats. Representative western blot and quantitative analysis of TLR4 (A), MyD88 (B), NF-κB p65 (C), and COX-2 (D) in different groups (*n* = 3). Data are presented as mean ± SEM. *p < 0.05, **p < 0.01 *vs.* Sham; ^#^*p* < 0.05, ^##^*p* < 0.01 *vs.* MCAO.

Furthermore, similar results for the modulation of the key proteins in the TLR4 pathway were observed (Figure 9). PTH or TAK-242 treatment markedly decreased the protein levels of TLR4, MyD88, NF-κB p65, and COX-2 in the brain tissue of MCAO rats, and combined treatment with PTH and TAK-242 did not produce a significant synergistic action compared with treatment with PTH or TAK-242 alone (*p* > 0.05).

## Discussion

In this study, we found that PTH pre-treatment could improve neurological function, and alleviate infarct volume and brain injury after cerebral ischaemia-reperfusion injury in rats, which were consistent with our previous study (Zhang et al. 2018). More importantly, PTH significantly suppressed a variety of severe inflammatory responses such as reducing inflammatory mediator release and inhibiting microglia and astrocyte activations, and the underlying mechanism was partially related to the TLR4/NF-κB/MAPK pathway.

Ischaemic stroke is a complex pathophysiological process that is induced by many factors, among which inflammation plays a crucial role (Eltzschig and Eckle 2011). During ischaemia onset, resident microglia and astrocytes were activated, and multiple inflammatory factors including cytokines, chemokines and enzymes were excessively released, which not only aggravate brain injury but also influence brain repair (Esenwa and Elkind 2016; Shi et al. 2019). Therefore, suppressing neuroinflammation and related factors could be a critical therapeutic approach for preventing and treating ischaemic stroke. In the current study, we observed that PTH significantly inhibit the activation of microglia and astrocytes in MCAO rats, as indicated by the reduced levels of Iba-1 and GFAP. In addition, PTH not only effectively inhibited the releases of three proinflammatory cytokines (IL-1β, IL-6 and TNF-α) and one chemokine (MCP-1), but also decreased the levels of two enzymes (iNOS and COX-2) in the brain. These data allude to the fact that PTH possess a comprehensive anti-neuroinflammatory potential in ischaemic stroke.

TLR4, one of the important innate and adaptive immune cell receptors, has been increasingly considered as a key target in regulating cerebral ischaemic injury-induced inflammation (Hakimizadeh et al. 2016; Zhang et al. 2017). TLR4 is stimulated during ischaemia occurrence and further activates the recruitment of MyD88. Subsequently, two parallel signalling pathways including NF-κB and MAPK were activated to induce the release of inflammatory mediators (Takaoka et al. 2005; Ling et al. 2018). Several previous studies have demonstrated the anti-inflammatory effect of PTH in hepatitis and arthritis, and NF-κB signalling is the current hotspot (Zheng et al. 2019; Deng et al. 2020). However, few studies concentrated on the anti-inflammatory effect of PTH in the TLR4 signalling pathway after ischaemic stroke. Therefore, we determined the TLR4 and its related downstream pathways in this study. Our results showed that the protein levels of TLR4, MyD88, and TRAF6 in MCAO rats were significantly elevated and that PTH effectively inhibited the up-regulation of these proteins, indicating that the TLR4 signalling pathway may participate in this effect. Many studies suggested that TLR4-mediated NF-κB activation is of great significance to regulating inflammatory mediators related with cerebral ischaemic injury, and its inhibition could reduce infarct volume and improve neurological function in MCAO mice (Li et al. 2013). In addition, ERK1/2, p38 and JNK in the MAPK signalling pathway were activated by TLR4, and the inhibitions of these three subgroups could produce a potential neuro-protective effect in ischaemic stroke (Tse et al. 2014). In this study, we found that PTH significantly decreased the p65 level and increased the IκBα level, and remarkably decreased the nucleus/cytoplasm ratio of NF-κB p65 in MCAO rats. Meanwhile, PTH also significantly decreased the protein levels of p-ERK1/2, p-p38, and p-JNK. All these indicated that the NF-κB and MAPK signalling pathways, the two important downstream signal pathways of TLR4, are also involved in the anti-inflammatory effect of PTH.

In addition, the TLR4 small-molecule inhibitor TAK-242 was used in this study to further investigate whether the TLR4 pathway mediated the anti-inflammatory effects of PTH. The results exhibited that combined treatment with PTH and TAK-242 significantly down-regulated the protein levels of TLR4, MyD88, NF-κB p65, and COX-2; inhibited the releases of IL-1β, IL-6, TNF-α, and MCP-1; alleviated cerebral infarct volume; and improved neurological deficits in MCAO rats. However, these alterations were not significantly different from those induced by individual treatment with PTH or TAK-242. Collectively, these results strongly suggested that the TLR4/NF-κB/MAPK signalling pathway was involved in the anti-inflammatory effects of PTH in ischaemic stroke.

Despite the above these findings, this study has some limitations. First, inflammatory response is a persistent feature throughout the progression of ischaemic stroke. However, our study only investigated the short-term protective effects and potential mechanism of PTH. Thus, a long-term study on PTH (lasting for more than 3 days at least) is necessary in the future. Second, PTH is composed of variety of active compounds with different contents, among which many compounds have diverse pharmacological effects and different molecular targets. Therefore, we believe that the protective effects of PTH should be mediated by more than one molecular target, and additional integrated strategies, such as metabolomics and network pharmacology combined investigation, are warranted.

## Conclusions

This study demonstrated that PTH could inhibit neuroinflammation, improve neurological function, and alleviate brain injury in MCAO rats. The potential mechanism of these effects was closely related to the TLR4/NF-κB/MAPK signalling pathway. This study provides fundamental evidence for the potential of PTH as an anti-inflammatory agent against ischaemic stroke and will help guide its clinical application.
